# The Antioxidant Activity of Limonene Counteracts Neurotoxicity Triggered byAβ_1-42_ Oligomers in Primary Cortical Neurons

**DOI:** 10.3390/antiox10060937

**Published:** 2021-06-09

**Authors:** Ilaria Piccialli, Valentina Tedeschi, Lucia Caputo, Giuseppe Amato, Laura De Martino, Vincenzo De Feo, Agnese Secondo, Anna Pannaccione

**Affiliations:** 1 Department of Neuroscience, Division of Pharmacology, Reproductive and Odontostomatological Sciences, School of Medicine, “Federico II” University of Naples, 80131 Naples, Italy; ilaria.piccialli@unina.it (I.P.); valentina.tedeschi@unina.it (V.T.); 2 Department of Pharmacy, University of Salerno, Via Giovanni Paolo II, 132, 84084 Fisciano, Italy; lcaputo@unisa.it (L.C.); g.amato29@studenti.unisa.it (G.A.); ldemartino@unisa.it (L.D.M.); defeo@unisa.it (V.D.F.)

**Keywords:** Alzheimer’s disease, Amyloid-β oligomers, limonene, antioxidant activity, potassium channels, ROS, acetylcholinesterase, neuroprotection

## Abstract

Many natural-derived compounds, including the essential oils from plants, are investigated to find new potential protective agents in several neurodegenerative disorders such as Alzheimer’s disease (AD). In the present study, we tested the neuroprotective effect of limonene, one of the main components of the genus *Citrus*, against the neurotoxicity elicited by Aβ_1-42_ oligomers, currently considered a triggering factor in AD. To this aim, we assessed the acetylcholinesterase activity by Ellman’s colorimetric method, the mitochondrial dehydrogenase activity by MTT assay, the nuclear morphology by Hoechst 33258, the generation of reactive oxygen species (ROS) by DCFH-DA fluorescent dye, and the electrophysiological activity of K_V_3.4 potassium channel subunits by patch-clamp electrophysiology. Interestingly, the monoterpene limonene showed a specific activity against acetylcholinesterase with an IC_50_ almost comparable to that of galantamine, used as positive control. Moreover, at the concentration of 10 µg/mL, limonene counteracted the increase of ROS production triggered by Aβ_1-42_ oligomers, thus preventing the upregulation of K_V_3.4 activity. This, in turn, prevented cell death in primary cortical neurons, showing an interesting neuroprotective profile against Aβ_1-42_-induced toxicity. Collectively, the present results showed that the antioxidant properties of the main component of the genus *Citrus*, limonene, may be useful to prevent neuronal suffering induced by Aβ_1-42_ oligomers preventing the hyperactivity of K_V_3.4.

## 1. Introduction

Alzheimer’s disease (AD) is a complex, multifarious syndrome characterized by the progressive loss of episodic memory and cognitive abilities [1]. Intracellular and extracellular deposits of the amyloid-β (Aβ) peptide play a key role in AD pathology [2]. Accumulating evidence supporting the amyloid cascade hypothesis shows that Aβ oligomers intervene in different pathways leading to AD neurodegeneration including synaptic dysfunction and neuronal network disruption [3]. Besides the well-known neurotoxic role of Aβ *per se*, the amyloidogenic protein transthyretin intervenes in the aggregation of amyloid fibrils, thus modulating its function overall [4]. At the molecular level, Aβ oligomers affect neuronal and glial cell functions by inducing unregulated production of reactive oxygen species (ROS) and subsequent oxidative stress, aberrant Ca^2+^ signaling, abnormal neuronal electrical activity, mitochondrial damage, endoplasmic reticulum (ER) stress, and apoptosis [5,6,7,8,9].

Despite impressive efforts to find new drugs, there is currently no cure for AD. Only two classes of drugs, namely cholinesterase inhibitors and the N-methyl-D-aspartate (NMDA) receptor antagonist memantine, are currently included in AD therapy [10,11]. However, their effects, which only target cognitive symptomatology, are moderate and unable to slow down AD progression.

Medicinal plants, already used in traditional medicine as alternative or complementary therapy for different pathological conditions, represent an important resource for the research of new therapeutic strategies in the treatment of AD as well as of other neurodegenerative disorders [12]. Of note, resveratrol, curcumin, ginsenoside, and many other natural compounds displayed significant neuroprotective effects. Among these compounds, monoterpenes, which are present as major components in many essential oils (EOs) from aromatic plants, display a wide range of biological features, including antioxidant, anti-inflammatory, and protective activities [13,14]. However, the therapeutic effects of monoterpenes in AD models need to be further investigated.

Limonene, a common monoterpene found as major component of the active complex of the genus *Citrus* [15], has been shown to exert anxiolytic, antinociceptive, antioxidant, and anti-inflammatory activity [16,17,18,19,20], as well as to display a protective effect against metabolic syndromes and gastrointestinal and respiratory tract diseases [21,22]. Interestingly, limonene has been suggested to act on the central nervous system, affecting the expression of adenylate cyclase 1 [15], which has been demonstrated to regulate cAMP levels. Many reports also showed that limonene promotes neural differentiation triggering neurite growth via p38/MAPK pathway [23,24]. Importantly, antioxidant and anti-inflammatory activities of limonene have been shown to be crucial for its protective action against Aβ_1-42_ toxicity [25,26]. In particular, recent studies reported that limonene is able to exert a neuroprotective effect in a *Drosophila* model of AD [26]. However, the exact mechanism underlying the effect of limonene in inhibiting Aβ_1-42_ -induced neurotoxicity remain to be clarified.

Several studies demonstrated that an imbalance of K^+^ concentrations associated with an inappropriate functioning of K^+^ channels in neuronal and glial cells is involved in AD pathophysiology [27,28,29]. In particular, increased K^+^ efflux and the subsequent reduction of cytoplasmic K^+^ concentrations trigger the activation of caspases and nucleases, thus inducing apoptosis [30], a process highly involved in AD neuronal loss. Moreover, K^+^ channels have also been involved in astroglial responses and neuroinflammation in AD [31,32,33,34]. Intriguingly, the fast inactivating K^+^ (I_A_) currents mediated through the voltage-gated K^+^ channel K_V_3.4 that contribute to the regulation of neuronal and astrocytic excitability have been implicated in AD pathology [29,32,35,36,37,38,39,40]. Previous studies by our group demonstrated that Aβ_1-42_ -induced up-regulation of K_V_3.4 is implicated in caspase-3 activation and in astrocytic responses to Aβ_1-42_ insult [32,35,36,38,39,40]. Of note, our previous investigations showed that the ROS signaling pathway induced by Aβ_1-42_ oligomers is an early biochemical event leading to the selective enhancement of K_V_3.4 currents through the activation of NF-kB transcriptional factor [36]. In addition, in other studies K_V_3.4 is reported to be an oxidation-sensitive channel since it is directly modulated by ROS increasing K_V_3.4 current amplitude [41]. Importantly, we observed that K_V_3.4 silencing or pharmacological inhibition with the sea anemone toxin blood depression substance-I (BDS-I) prevented Aβ_1-42_-induced insults in neurons as well as abnormal Ca^2+^ signaling and ER stress in astrocytes [36,40]. Strikingly, in vivo silencing of K_V_3.4 was able to reduce glial fibrillary acidic protein (GFAP) over-expression and Aβ_1-42_ trimer burden in the Tg2576 mice brain, a transgenic model of AD [32]. The up-regulation of K_V_3.4 in Aβ_1-42_-insults in both neurons and astrocytes has therefore emerged as an important mechanism to be investigated and a new possible pharmacological target in AD treatment.

In view of these considerations, the purpose of the present study has been to investigate the effect of limonene, one of the main constituents of several plants from the genus *Citrus* on acetylcholinesterase (AChE) activity and its putative neuroprotective effect against Aβ_1-42_ neurotoxicity in an in vitro model of AD, namely rat primary cortical neurons exposed to oligomeric species of the neurotoxic Aβ_1-42_ peptide. In particular, we assessed the ability of limonene to counteract the effect of Aβ_1-42_ oligomers on neuronal viability, ROS production, and K_V_3.4-mediated I_A_ currents.

## 2. Materials and Methods

### 2.1. Chemicals and Reagents

EO of *Citrus medica cv rugosa*, limonene (sum of enantiomers, purity > 98%), 2′,7′-dichlorodihydrofluorescein diacetate (DCFH-DA), poly-L-lysine, and fluorescent DNA-binding dye bis-Benzimide H 33258 (Hoechst-33258), nimodipine, and 3[4,5-dimethylthiazol-2-yl]-2,5-diphenyl-tetrazolium bromide (MTT) were purchased from Sigma-Aldrich (St. Louis, MO, USA). Biochemical cck-8 kit for WST-8 assay was purchased from Dojindo (Kumamoto, Japan). RPMI 1640 medium, fetal bovine serum (FBS), horse serum (HS), non-essential amino acids, penicillin, streptomycin, and PBS were from Gibco-BRL (Grand Island, NY, USA). Nerve growth factor (NGF) and tetrodotoxin (TTX) were from Alomone Lab (Jerusalem, Israel). The Aβ_1-42_ peptide (> 95% pure) was synthesized by INBIOS (Pozzuoli, Naples, Italy) using the Aβ_1-42_ sequence of human APP [UniProtKB-P05067 (A4_HUMAN)].

### 2.2. In Vitro Anti-Acetylcholinesterase Activity

AChE inhibitory activity assay was performed according to a previously described spectrophotometric method [42]. Absorbance was measured at 405 nm in a spectrophotometer (Thermo Scientific Multiskan GO, Monza, Italy). Galantamine was used as positive control and bidistillated water as a negative control. The percentage inhibition of AChE activity was calculated by comparison with the negative control using the following equation: AChE inhibition % = [(A_0_ − A_1_)/A_0_]*100, where A_0_ is the absorbance of the control without sample and A_1_ is the absorbance of the sample.

### 2.3. Cell Cultures

Rat pheochromocytoma (PC12) cells were cultured as previously described [35]. Neuronal differentiation was obtained by exposing these cells to nerve growth factor (NGF; 50 ng/mL) for 7 days [35,36]. Then, NGF-differentiated cells were seeded on 96-well plates and used after 7 days. Human neuroblastoma (SH-SY5Y) cells were cultured as previously described [43].

### 2.4. Primary Cortical Neurons

Cortical neurons were obtained from brains of 14/16-day-old Wistar rat embryos and dissected as reported previously [44]. Neurons were cultured in a humidified 5% CO_2_ atmosphere, and the culture medium was changed every 2 days. For microfluorimetric and electrophysiological studies, cells were seeded on glass coverslips (Fisher, Springfield, NJ, USA) coated with poly-D-lysine and used at least 12 h after seeding. Italian Ministry of Health and the local Animal Care Committee of “Federico II” University of Naples (Italy) approved all animal procedures adopted (D. Lgs. 4th March 2014 from Italian Ministry of Health; DIR 210/63 UE; 12/2018-UT7).

### 2.5. Aβ Treatment

Aβ_1-42_ oligomers were re-suspended as previously described [38]. Aβ_1-42_ was added to culture medium at the final concentration of 5 μM for 24 h. The pre-aggregated preparation of Aβ oligomers was analyzed in SDS-PAGE using a rabbit monoclonal Aβ antibody (D54D2) (Cell signaling, MA, USA) on precast gels 4–20% [32].

### 2.6. MTT Assay

Mitochondrial dehydrogenase activity was assessed by the MTT assay as previously described [45]. Data are expressed as a percentage of cell viability compared to control cultures.

### 2.7. Assessment of Intracellular ROS Production

Cortical neurons plated on glass coverslips were exposed to limonene in the presence or in absence of Aβ_1-42_ oligomers for 24 h. At the end of the treatment, cells were incubated with a physiological solution containing DCFH-DA (17.5 µM) [35,46]. Cells were washed with a stopping solution containing EGTA. Each coverslip was rapidly placed into a perfusion chamber (Medical System, Co. Greenvale, NY, USA) and acquired with the Zeiss Axiovert 200 microscope (Carl Zeiss, Germany) equipped with MicroMax 512BFT cooled CCD camera (Princeton Instruments, Trenton, NJ, USA). Using a 40X objective, each coverslip was exposed at 485-nm excitation for 10 s and the emitted light was passed through a 530-nm barrier filter.

### 2.8. Electrophysiology

K^+^ currents were recorded from primary rat cortical neurons using a commercially available amplifier (Axopatch 200B, Axon Instruments, Union City, CA, USA), as previously described [35,36]. Currents were filtered at 5 kHz and digitized using a Digidata 1322A interface (Molecular Devices, CA, USA). Data were acquired and analyzed using pClamp software (version 9.0, Molecular Devices, CA, USA). The pipette solution contained the following (in mM): 100 K-gluconate, 20 NaCl, 1 Mg-ATP, 0.1 CaCl_2_, 2 MgCl_2_, 0.75 EGTA, and 10 HEPES, adjusted at pH 7.4 with KOH. The extracellular solution contained the following (in mM): 126 NaCl, 1.2 NaHPO_4_, 2.4 KCl, 2.4 CaCl_2_, 1.2 MgCl_2_, 10 glucose, and 18 NaHCO_3_, pH 7.4. TTX (50 nM) and nimodipine (10 µM) were added to extracellular solution to inhibit Na^+^ and Ca^2+^ currents. The K^+^ current components (inactivating component I_A_ and delayed-rectifier non-inactivating component I_DR_) were discriminated using the appropriated electrophysiological protocols as previously described [35,36]. Possible changes in cell size occurring upon specific pharmacological treatments were calculated by monitoring the capacitance of each cell membrane, which is directly related to membrane surface area, and the current amplitude was expressed as current densities (pA/pF) as previously described [35,36].

### 2.9. Assessment of Nuclear Morphology

Nuclear morphology was studied by Hoechst-33258 as previously described [35] Cells were fixed in 4% paraformaldehyde and then incubated with Hoechst 33258 (1 µg/mL/5 min/37 °C). Images were acquired with a CoolSnap camera (Media Cybernetics Inc, Silver Spring, MD, USA) using the Nikon Eclipse E400 microscope (Nikon, Torrance, CA, USA). Image analysis was performed with the Image-Pro Plus 4.5 software (Media Cybernetics Inc, Silver Spring, MD, USA). A set of 330 nm/450 nm filters was used to detect Hoechst-33258. Pathological nuclei are characterized by chromatin condensation, fragmentation, and decrease in size. Values acquired in all conditions were expressed as percentage of total nuclei.

### 2.10. Western Blotting

SH-SY5Y cells were treated with EO of *Citrus* medica rugosa. After 24 h of treatment, cells were collected and lysed as previously described [43]. Nitrocellulose blots were incubated with primary anti-phosphoERK (pERK; Santa Cruz Biotechnology, Santa Cruz, CA, USA; sc-377400), anti-ERK (Santa Cruz Biotechnology, Santa Cruz, CA, USA, sc-271269), anti-PKA (Elabscience, USA) or anti-calregulin (Santa Cruz Biotechnology, Santa Cruz, CA, USA;sc-101436) for 3 h at room temperature and then with horseradish peroxidase-conjugated secondary antibody (Amersham Biosciences, Pittsburgh, PA, USA).

### 2.11. Statistics

**Figure 1:**
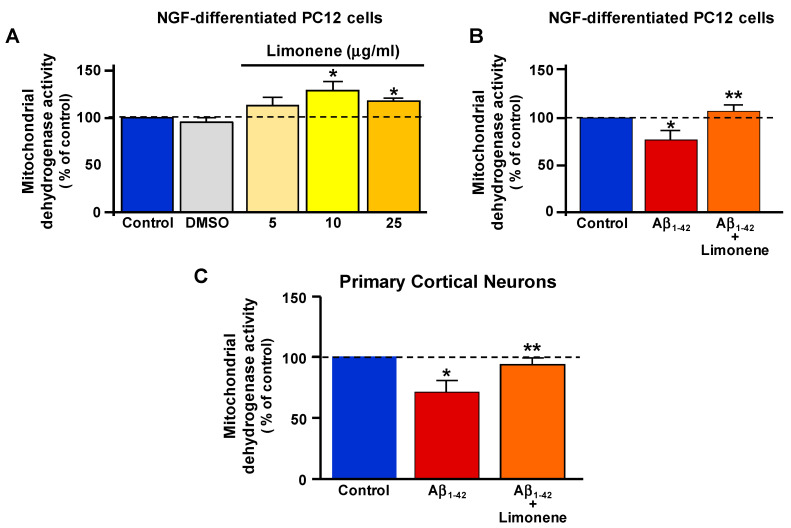
Effects of limonene on mitochondrial dehydrogenase activity in NGF-differentiated PC12 cells and primary cortical neurons exposed to Aβ_1-42_ oligomers. (**A**) Evaluation of the mitochondrial dehydrogenase activity by MTT assay in NGF-differentiated PC12 cells exposed to limonene at different concentrations (5, 10, and 25 µg/mL) for 24 h. (**B**,**C**) Quantification of mitochondrial dehydrogenase activity assessed by MTT assay in NGF-differentiated PC12 cells (**B**) and primary cortical neurons (**C**) exposed to Aβ_1-42_ oligomers (5 µM/24 h) in the presence and in absence of limonene (10 µg/mL, 30 min pre-treatment). Data are shown as percentage of mitochondrial dehydrogenase activity (compared to control cells) and values are expressed as mean ± SEM of three independent experimental sessions (* *p* < 0.05 vs control; ** *p* < 0.05 vs. Aβ_1-42_).

**Figure 2:**
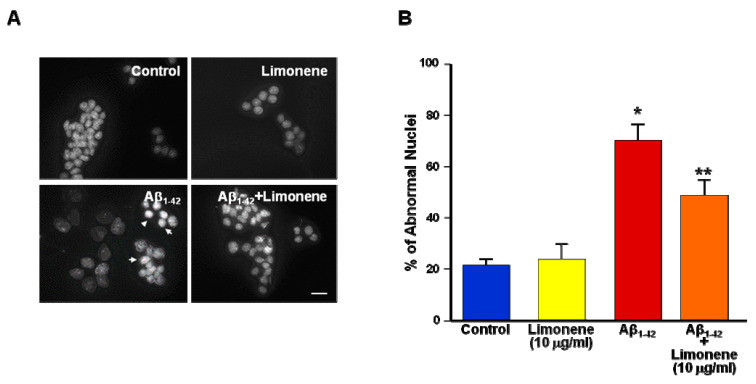
Effects of limonene on nuclear morphology in primary cortical neurons exposed to Aβ_1-42_ oligomers. (**A**) Representative images of Hoechst-33258 nuclear morphological abnormalities in primary cortical neurons under control conditions, in neurons treated with limonene (10 µg/mL/24 h), and treated with Aβ_1-42_ oligomers (5 µM/24 h; see white arrows) in the absence and in presence of limonene (10 µg/mL, 30 min pre-treatment) Scale bar: 20 μm. (**B**) Quantification of nuclear morphology with Hoechst-33258 in A. Data are shown as percentage of abnormal nuclei (relative to total nuclei) and values are expressed as mean ± SEM of four independent experimental sessions in which at least 10 microscopic fields were analyzed (* *p* < 0.05 vs. control; ** *p* < 0.05 vs. Aβ_1-42_).

**Figure 3:**
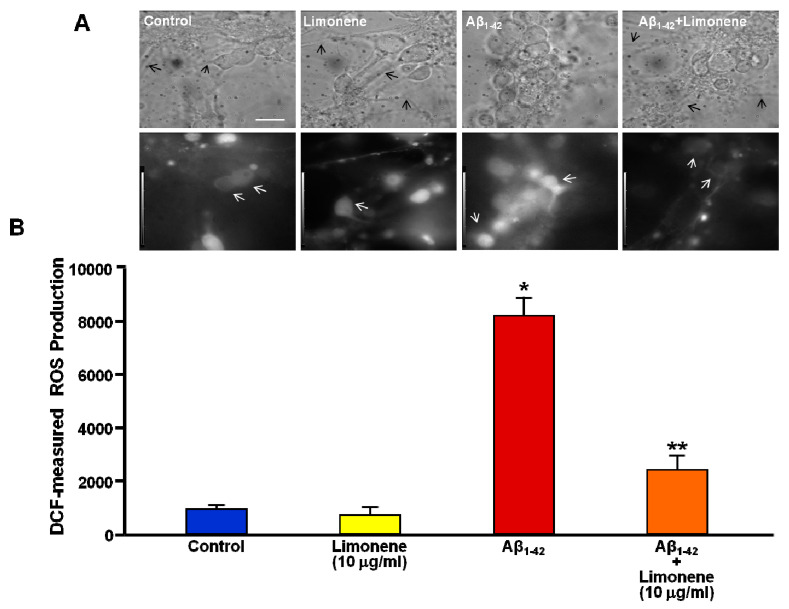
Effects of limonene on ROS production in primary rat cortical neurons exposed to Aβ_1-42_ oligomers. (**A**) Representative phase-contrast (top) and corresponding DCF fluorescence images (bottom) of primary cortical neurons under control conditions, in neurons treated with limonene (10 µg/mL/24 h), or treated with Aβ_1-42_ oligomers (5 µM/24 h) in the absence and in presence of limonene (10 µg/mL, 30 min pre-treatment). Black and white arrows indicate differences occurring at the level of prolongations and fluorescence intensity, respectively. Scale bar: 20 μm. (**B**) Quantification of intracellular ROS production in A. Data are depicted as DCF fluorescence values (arbitrary units) expressed as mean ± SEM of three independent experimental sessions. * *p* < 0.05 vs controls; ** *p* < 0.05 vs Aβ_1-42_.

**Figure 4:**
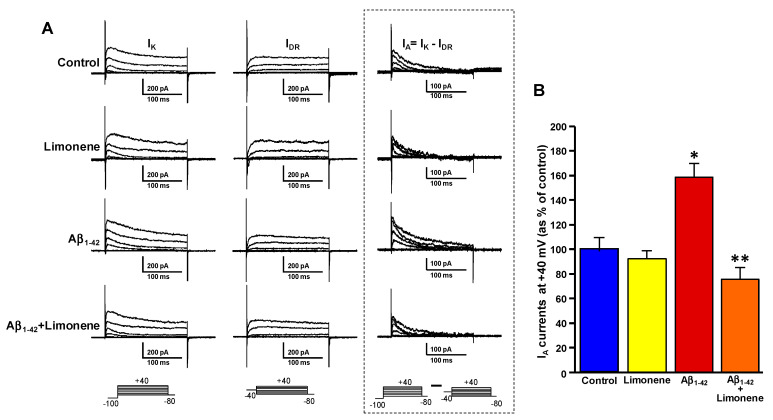
Effects of limonene on K_V_3.4 channels in primary rat cortical neurons exposed to Aβ_1-42_ oligomers. (**A**) Representative outward K^+^ currents recorded in primary cortical neurons under control conditions, in neurons treated with limonene (10 µg/mL/24 h), and treated with Aβ_1-42_ oligomers (5 µM/24 h) in the absence and in presence of limonene (10 µg/mL, 30 min pre-treatment). On the left, total K^+^ currents (I_K_) elicited by depolarizing steps starting from −100 mV of increasing voltages are shown. On the middle, the delayed K^+^ currents (I_DR_), obtained in the same neurons and elicited by depolarizing steps starting from −40 mV of increasing voltages are shown. On the right, fast inactivating currents (I_A_) carried by K_V_3.4, obtained in each cell upon subtraction I_K_-I_DR_, are shown. (**B**) Quantification of I_A_ in (**A**). The peak values of I_A_, measured at the beginning of the +40 mV depolarizing pulse, are expressed as percentage mean ± SEM of three independent experimental preparations (*n* = 12 neurons for each group). * *p* < 0.05 vs controls; ** *p* < 0.05 vs Aβ_1-42_.

GraphPad Prism 6.02 was used for statistical analyses (GraphPad Software, La Jolla, CA, USA). Data are expressed as the mean ± SEM (Figure 1, Figure 2, Figure 3 and Figure 4) or mean ± SD (Figure S1) of the values obtained from individual experiments. Statistical comparisons between groups were performed by one-way analysis of variance (ANOVA) followed by the Newman–Keuls’ test; *p* < 0.05 was considered significant.

## 3. Results

### 3.1. Effect of Limonene on Acetylcholinesterase Activity

AChE, the enzyme involved in the hydrolysis of acetylcholine, plays an important role in the neurodegeneration occurring in AD. The AChE inhibitors are currently included in AD therapy since they display efficacy in relieving cognitive symptoms in AD patients [10,11]. Interestingly, limonene showed a significant activity against acetylcholinesterase with a calculated IC_50_ of 7.7 µg/mL that was measured in vitro by the Ellman’s colorimetric method (Table 1). Of note, the reported IC_50_ for limonene activity is almost comparable to that of galantamine, which has been used as positive control (Table 1).

### 3.2. Effect of Limonene on Mitochondrial Dehydrogenase Activity Reduction Induced by Aβ_1-42_ Oligomers in Primary Cortical Neurons

Before investigating the possible neuroprotective effect of limonene against Aβ_1-42_-induced neurotoxicity, we assessed mitochondrial dehydrogenase activity of NGF-differentiated PC12 cells exposed to different concentrations of limonene (5, 10, and 25 µg/mL/24 h) in order to exclude any putative cytotoxicity of the compound as well as to identify the appropriate concentration for subsequent experiments. Importantly, NGF-differentiated PC12 cells treated for 24 h with limonene did not display any significant reduction but rather a moderate increase in mitochondrial dehydrogenase activity. In fact, at the concentration of 10 µg/mL and 25 µg/mL respectively, it showed a significant ability to increase mitochondrial dehydrogenase activity (Figure 1A). Of note, the concentration of 10 µg/mL was very similar to the IC_50_ calculated for the inhibition of AChE by galantamine used as positive control (see Table 1). Therefore, the putative neuroprotective effect of 10 µg/ml limonene was investigated in NGF-differentiated PC12 cells and in primary cortical neurons exposed to Aβ_1-42_ oligomers (5 µM/24 h) (Figure 1B,C). In particular, NGF-differentiated PC12 cells and neurons were pre-incubated with 10 µg/mL limonene 30 min before the exposure to Aβ_1-42_ oligomers. After 24 h of incubation with Aβ_1-42_ oligomers, mitochondrial dehydrogenase activity was assessed. Both NGF-differentiated PC12 cells and primary cortical neurons treated with 5 µM Aβ_1-42_ oligomers alone displayed a significant reduction in mitochondrial dehydrogenase activity in comparison to untreated cells. By contrast, the reduction of mitochondrial dehydrogenase activity was prevented in NGF-differentiated PC12 cells and primary cortical neurons pre-treated with 10 µg/mL limonene (Figure 1B,C). From a transductional point of view, the EO of *Citrus medica cv. ‘rugosa’* containing 67% of limonene [15] produced a significant downregulation of pERK and PKA expression in SH-SY5Y cells (Figure S1).

### 3.3. Effect of Limonene on Nuclear Morphology Alteration Induced by Aβ_1-42_ Oligomers in Primary Cortical Neurons

To further study the neuroprotective effect of limonene against Aβ_1-42_ toxicity, we also performed labeling experiments with the fluorescent DNA binding dye Hoechst- 33258 on primary cortical neurons treated with Aβ_1-42_ oligomers (5 µM/24 h) in the presence and in absence of limonene. In accordance with the reduction of mitochondrial dehydrogenase activity, nuclear morphological assessment revealed a marked pyknosis, fragmentation, and decrease in size in neurons exposed to Aβ_1-42_ oligomers compared to untreated neurons (Figure 2 and Figure S2). On the other hand, 30 min of pre-treatment with 10 µg/mL limonene was able to significantly counteract the alteration of nuclear morphology induced by Aβ_1-42_ oligomers (Figure 2 and Figure S2).

### 3.4. Effect of Limonene on ROS Production Induced by Aβ_1-42_ Oligomers in Primary Cortical Neurons

A great amount of studies suggested that oxidative stress associated with increased ROS production may constitute an upstream event in AD pathogenesis. Previous studies by our group showed that Aβ_1-42_ oligomers at the concentration of 5 µM induce an increase of ROS production that peaks at 3 h and lasts for 24 h in both NGF-differentiated PC12 cells and primary hippocampal neurons [35,36]. Therefore, the generation of ROS was detected by DCFH-DA fluorescent dye in primary cortical neurons exposed to Aβ_1-42_ oligomers (5 µM/24 h) in the presence and in absence of 10 µg/mL limonene. In line with our previous observations, primary cortical neurons treated with Aβ_1-42_ oligomers displayed increased DCF-monitored fluorescent intensity indicating a significant increase of ROS production compared with untreated neurons (Figure 3 and Figure S3). Importantly, the pre-treatment with limonene at the concentration of 10 µg/mL prevented the significant over-production of ROS induced by Aβ_1-42_ oligomers, as indicated by a decrease in DCF-monitored fluorescent intensity compared with Aβ_1-42_-treated neurons (Figure 3 and Figure S3).

### 3.5. Effect of Limonene on the Upregulation of Fast-Inactivating I_A_ Currents Triggered by Aβ_1-42_ Oligomers in Primary Cortical Neurons

Previously, we provided evidence that Aβ_1-42_ oligomers induced a selective up-regulation of K_V_3.4 channels through the ROS-dependent activation of the transcription factor NF-kB [36] and that the subsequent increase of K^+^ efflux was involved in neuronal and astrocytic damage [32,36,40]. Since the blockade of K_V_3.4 appeared to be an effective strategy to counteract Aβ_1-42_-mediated caspase-3 overactivation [38,39], we here tested the hypothesis that limonene could prevent the ROS-dependent up-regulation of fast-inactivating I_A_ currents mediated by K_V_3.4 in primary cortical neurons exposed to Aβ_1-42_ oligomers. First, we performed patch-clamp experiments in primary cortical neurons treated with Aβ_1-42_ oligomers (5 µM/24 h) to assess fast-inactivating I_A_ current amplitude carried by K_V_3.4 channels. In line with our previous reports, patch-clamp experiments revealed that Aβ_1-42_ oligomers were able to markedly enhance I_A_ density. On the other hand, we found that pre-treatment with 10 µg/mL limonene largely prevented the increase of fast-inactivating I_A_ currents induced by Aβ_1-42_ oligomers (Figure 4).

## 4. Discussion

**Scheme 1:**
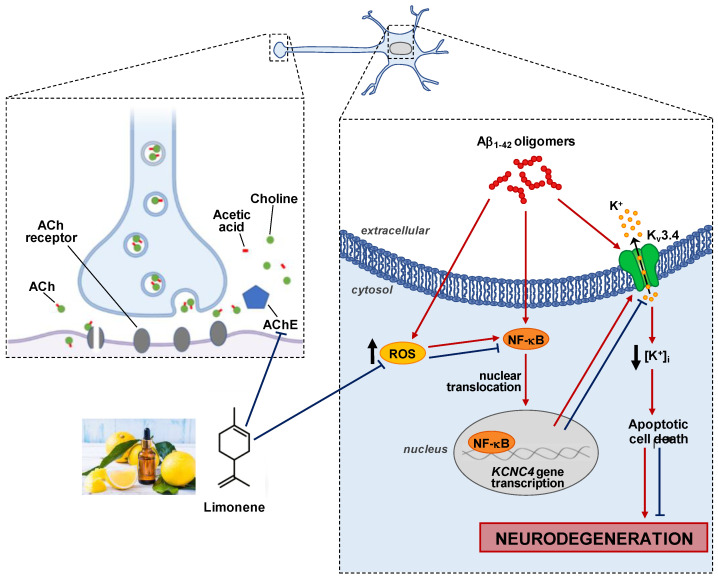
Scheme of the putative mechanism of limonene action in cortical neurons exposed to Aβ_1-42_ oligomers.

In the present study, we evaluated the neuroprotective effects of the monoterpene limonene, the main constituent of plants from *Citrus* genus, against AD in an in vitro model of the disease represented by primary cortical neurons exposed to Aβ_1-42_ oligomers. The results obtained suggested that limonene was able to protect primary cortical neurons from cell damage induced by Aβ_1-42_ oligomers by preventing ROS production and K_V_3.4 channel hyperfunction (Scheme 1).

Molecularly, limonene showed a specific activity against acetylcholinesterase almost comparable to galantamine, a well-known drug used in AD therapy. Moreover, limonene was able to prevent Aβ_1-42_ oligomer-induced decrease in mitochondrial dehydrogenase activity and increase in ROS production, thus exerting a neuroprotective effect in primary cortical neurons exposed to Aβ_1-42_ oligomers. Our findings are in accordance with a previous in vivo study showing that limonene may exert a neuroprotective effect against the toxicity of Aβ_1-42_ in a *Drosophila* AD model through a strong antioxidant action [26].

In the present study we showed that limonene, acting on ROS production, prevented the K_V_3.4 current enhancement induced by Aβ_1-42_ oligomers. Of note, the increase in ROS level observed in AD is recognized to be an early biochemical event leading to the enhancement of K_V_3.4 currents induced by Aβ_1-42_ oligomers [32,36,40]. Therefore, consistent with our previous results, we hypothesized that a marked increase in ROS levels observed here may produce the upregulation of K_V_3.4 activity also in primary cortical neurons. This result bears striking homology with previous data showing that the antioxidant action of Vitamin E is able to prevent the upregulation of K_V_3.4 channel activity induced by Aβ_1-42_ oligomers in neurons [35]. Moreover, it has been shown that the increased expression and function of K_V_3.4 following Aβ_1-42_ oligomers exposure are critically dependent on Ca^2+^-induced increase in ROS production, which in turn prompts K_V_3.4 transcriptional activation through a nuclear factor κB-dependent (NF-κB) pathway [35,36]. Remarkably, NF-κB was the first transcription factor shown to be redox-regulated [47,48].

Of note, limonene is able to decrease NF-κB nuclear activation via AMP-activated protein kinase phosphorylation [49]. Therefore, the blockade of ROS-induced NF-κB activation could be involved in the neuroprotective mechanism elicited by limonene. However, a direct ROS scavenging action of the natural compound in the present AD model cannot be ruled out.

On the other hand, the important involvement of K_V_3.4 channels in the Aβ_1-42_ neurotoxicity is further supported by the results showing that BDS-I, a K_V_3.4 blocker [50], may exert a potent neuroprotective action both in AD neurons and astrocytes exposed to Aβ_1-42_ oligomers [36,40].

In respect to the effect of limonene on ROS production, these species seem to play a relevant role in the neurotoxic cascade of Aβ_1-42_. In fact, the transient influx of Ca^2+^ ions induced by Aβ_1-42_ oligomers may trigger intracellular cascades that lead not only to increased levels of ROS but also to simultaneous mitochondrial functional impairment characterized by activation of the permeability transition pore in the inner mitochondrial membrane, cytochrome c release, and depletion of ATP [35]. In this regard, it has been demonstrated that the blockade of K_V_3.4 may inhibit MPP+-induced cytochrome c release from the mitochondrial intermembrane space to the cytosol and mitochondrial membrane potential depolarization [41].

Another important neuroprotective process modulated by limonene is autophagy. Interestingly, limonene stimulates the autophagic flux through a rapid ERK activation [51]. Of note, the pharmacological inhibition of Kv3.4 by BDS-I counteracts intracellular pH regulation and ERK activation in A549 cells [52], thus further supporting the transduction modulation of K_V_3.4 channel by limonene. Besides a plethora of functions mediated at cellular and subcellular level, the ERK1/2 transduction element may negatively regulate the expression of β-secretase, the proteolytical enzyme mainly involved in the production of the neurotoxic Aβ_1-42_ peptide [53]. On the other hand, several studies provide direct evidence on the possible involvement of MAP kinase pathway in the hyper-phosphorylation of tau underlining the role played by ERK1/2 activation in the Aβ_1-42_ deposition during AD [54]. In addition, targeting ERK1/2 activity may slow tau spreading in sporadic AD thus offering a new putative neuroprotective strategy in the major form of AD [55]. In accordance to the latter study, our preliminary data (Figure S1) suggested that the EO *Citrus medica cv rugosa*, containing high levels of limonene, reduced ERK1/2 activation.

Another aspect that deserves attention is the putative clinical relevance of the present data. In fact, the use of essential oil containing limonene, or limonene alone, would be desirable in the therapy of AD symptoms, which is in line with our results showing the antioxidant properties of the natural compound and considering its ability to counteract Aβ_1-42_-induced K_V_3.4 hyperfunctionality in cortical neurons. Of course, our in vitro results should be reproduced in vivo to prove not only the efficacy of the treatment in a more complex model of the disease but also to define the pharmacokinetic profile of limonene. However, in line with our in vitro data, a recent manuscript shows a significant cognitive-enhancing effect of essential oil containing limonene in a scopolamine-induced amnesia model [56]. Interestingly, the authors correlate this therapeutic effect to the essential oil ability in inhibiting acetyl/butirrylcholinesterase activities [56]. Although our results are in line with this recent manuscript, we additionally demonstrated that limonene alone may exert the same AChE inhibitory activity than the essential oil containing other components. However, it would be desirable to go even further by performing in vivo experiments in AD transgenic mice to set up a therapeutic window of limonene and to study its protective effect in a more complex model.

Considering that ROS-mediated K_V_3.4 overexpression may intervene in both neurodegeneration and neuroinflammation underlying AD development [29,32,35,36,37,38,39,40], limonene may assume a novel neuroprotective meaning. Therefore, limonene, controlling the modulation of K_V_3.4 channels in AD brain via ROS production, might represent a novel therapeutic approach for slowing down the progression of the disease. Therefore, after an accurate examination of the molecular pathway involved in its mechanism, the modification of limonene to serve as prominent scaffold in designing novel bioactive compounds should be taken into consideration as a new potential avenue in AD intervention.

## 5. Conclusions

In this manuscript we showed that limonene exerts a novel neuroprotective effect in AD. In particular, limonene, controlling the modulation of K_V_3.4 channels via ROS level reduction, might represent a novel therapeutic approach for slowing down the progression of the disease. Moreover, limonene displays a specific activity against AChE almost comparable to galantamine, a well-known drug used in AD therapy. In this respect, the involvement of AChE metabolic activity in Aβ fibril formation is considered one of the most interesting future perspectives in AD therapy. For instance, AChE activity has been mainly associated with the amyloid core of senile plaques in the brain of AD patients [57,58]. Moreover, AChE activity increases and accelerates the aggregation of Aβ [59], as detected by thioflavin-T fluorescence assay [60]. Consequently, AChE inhibitors, such as donepezil and tacrine, reduce Aβ aggregation thus showing a certain therapeutic potential in AD [61]. Another important consideration is that limonene may exert a neuroprotective effect against Aβ toxicity through several molecular mechanisms including the inhibition of AChE, the antioxidant activity, the inhibition of K_V_3.4 hyperfunction and the downregulation of pERK. These mechanisms are not all simultaneously shared by the other AChE inhibitors. In fact, among the most studied drugs, tacrine and donepezil are the only two therapeutic compounds displaying some of the mechanisms displayed by limonene [61]. Therefore, limonene could represent an interesting multi-target molecule useful to design novel bioactive compounds slowing down AD progression.

## Figures and Tables

**Table 1:** Inhibitory effects of limonene on AChE activity. Concentration-dependent effect of limonene and galantamine against AChE activity measured in vitro (n.a. = not active).

Compound	Concentration (mg/mL)	AChE Inhibition (%)	IC50 (mg/mL)
Limonene	100	87.8 ± 1.4	
	10	78.4 ± 1.8	7.7 ± 1.2
	1	n.a	
Galantamine	100	100.1 ± 1.9	
	10	96.2 ± 2.1	
	1	85.0 ± 0.8	0.6 ± 0.2
	0.1	n.a	

## Data Availability

Not applicable.

## Supplementary Materials

The following are available online at https://www.mdpi.com/article/10.3390/antiox10060937/s1, Figure S1: Expression of pERK and PKA proteins in SH-SY5Y cells treated with *Citrus medica* cv *rugosa*; Figure S2: Nuclear morphology (images) of primary cortical neurons under control conditions, in the presence of limonene, Aβ_1-42_ or limonene + Aβ_1-42_; Figure S3: ROS production (images) in primary cortical neurons under control conditions, in the presence of limonene, Aβ_1-42_ or limonene + Aβ_1-42._
