# Supplementary material for: The Antioxidant Activity of Limonene Counteracts Neurotoxicity Triggered byAβ1-42 Oligomers in Primary Cortical Neurons
Source: Antioxidants (Basel). 2021 Jun 9;10(6):937. doi: 10.3390/antiox10060937 (PMC8227170; doi:10.3390/antiox10060937)
Supplement: Supplementary file 1 [file antioxidants-10-00937-s001.zip › antioxidants-1206156-supplementary.pdf]

antioxidants

MDPI

Supplementary Material

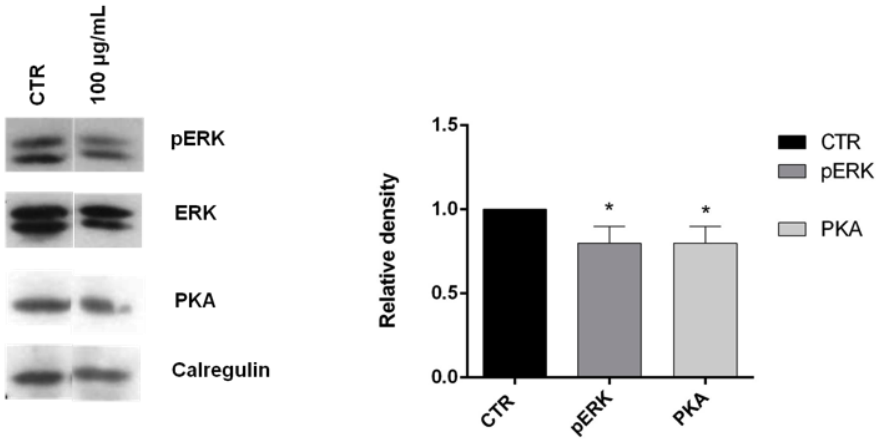

Figure S1 Relative expression levels of pERK and PKA proteins in SH-SY5Y cells treated with C. *medica* cv rugosa or with vehicle (CTR). Densitometric values of each protein have been normalized for calregulin values. The quantification of the effect of C. *medica* (100 µM) on pERK and PKA compared with CTR is reported on the right. Values are the mean ± SD of 3 experiments. * $p < 0.05$ vs CTR (ANOVA followed by Dunnett's multiple comparison test)

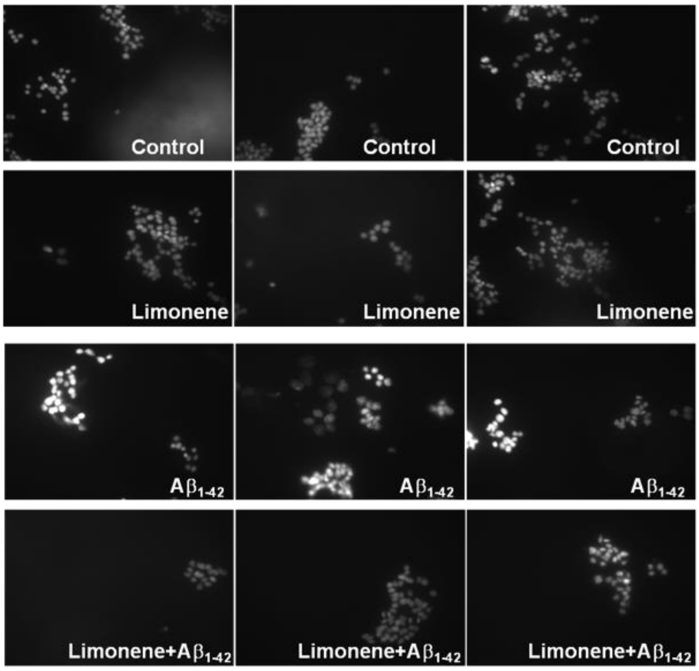

Figure S2 Representative Hoechst 33258-images from primary cortical neurons under control conditions, in the presence of limonene, $A\beta_{1-42}$ or limonene+$A\beta_{1-42}$.

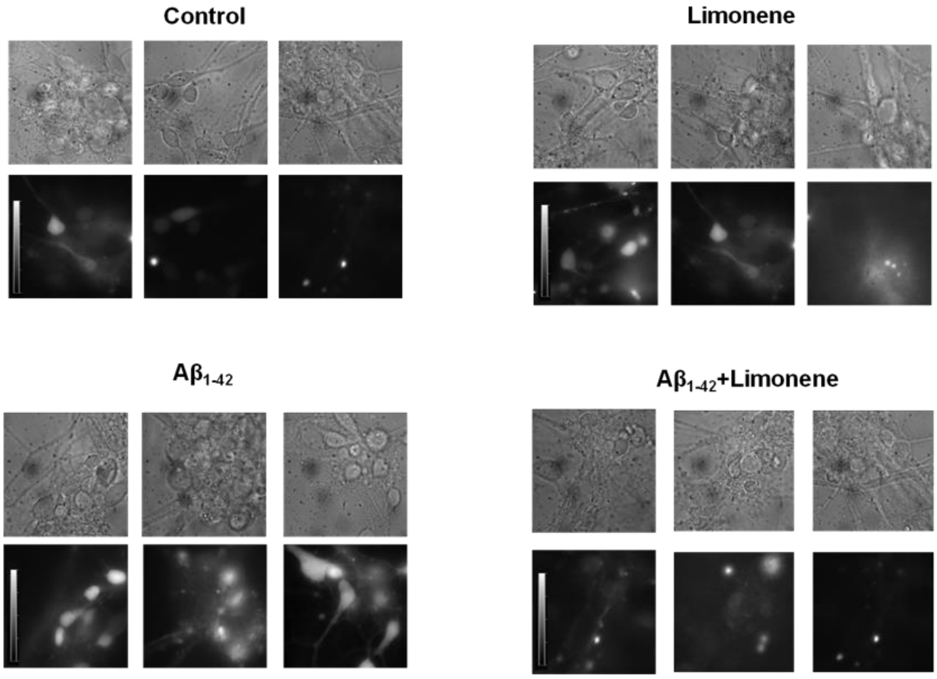

Figure S3 Representative DCF-images from primary cortical neurons under control conditions, in the presence of limonene, Aβ-1-42 or limonene+Aβ1-42
